# Supplementary material for: Active microbial communities and their extrachromosomal elements link organic matter degradation to methane cycling in anoxic sediments
Source: ISME Commun. 2026 Jul 26;6(1):ycag217. doi: 10.1093/ismeco/ycag217 (PMC13502194; doi:10.1093/ismeco/ycag217)
Supplement: Supplementary file 1 [file web_material_ycag217.zip › Supplementary_materials_ycag217.docx]

**Supplementary information**

**Active microbial communities and their extrachromosomal elements link organic matter degradation to methane cycling in anoxic sediments**

Bledina Dede^1^, Hanna Zehnle^1,#^, Emilie Skoog^1,2^, Taylor Priest^1,3^, Karin Beck^4^, Helmut Bürgmann^4^, and Marie C. Schoelmerich^1^

^1^ Department of Environmental Systems Science, Institute of Biogeochemistry and Pollutant Dynamics, ETH Zürich, 8092 Zürich, Switzerland

^2^ Scripps Institution of Oceanography, University of California, San Diego, La Jolla, CA, United States

^3^ Department of Biology, Institute of Microbiology, ETH Zürich, 8049 Zürich, Switzerland

^4^ Eawag, Swiss Federal Institute of Aquatic Science and Technology, 6047 Kastanienbaum, Switzerland

^#^ current affiliation: Facultad de Ciencias, Universidad de la República, Montevideo, Uruguay

**Methods**

**MAG reconstruction**

The trimmed reads were then mapped to the assemblies with BBMap v.39.01 using the parameter ambiguous=random. The resulting Sequence Alignment Map (SAM) files were converted to Binary Alignment Map (BAM) format using Samtools v.1.17 (1). BAM files were then sorted and indexed with Samtools (v.1.17). For each core, all trimmed reads from the six horizons were mapped to all six assemblies to obtain more coverage data for binning. For binning, three tools were used for each sample: MetaBAT2 v.2.17 (2), MaxBin2 v.2.2.7 (3), and CONCOCT v.1.1.0 (4). DAS_Tool v.1.1.7 (5) was used to extract the best bins from the three binning tools. Bins from all samples within each core were then pooled and dereplicated at species level (95% average nucleotide identity) using dRep v.3.4.5 (6), applying a minimum completeness of 50% and a maximum contamination of 10%, yielding medium- to high-quality metagenome-assembled genomes (MAGs) according to MiMAG standards (7) (**Table S4**).

MAGs were taxonomically classified using GTDB-Tk v.2.4.0 (8) and the GTDB database release 220 (**Table S5**). Additionally, GTDB-Tk was used for phylogenetic tree calculations of bacterial and archaeal MAGs. CoverM v.0.6.1 (9) was used to obtain relative abundances of the MAGs per sample. DRAM v.1.5.0 (10) and Metabolic v4.0 (11) was run on the final MAGs for functional annotation. Phylogenetic gene trees were calculated as described in (12).

**EcDNA identification, abundance, and activity analysis**

Viral relative abundance was estimated by mapping paired-end metagenomic reads from each depth independently to the dereplicated viral genomes using CoverM and requiring ≥95% nucleotide identity and ≥85% read alignment coverage. Coverage and abundance metrics, including mean coverage, covered fraction, relative abundance, and RPKM, were calculated. CoverM was also used to assess viral transcriptional activity by mapping metatranscriptomic reads to the same viral genome set using identical mapping thresholds and additionally reporting TPM values. Viral taxonomic identification was derived from geNomad taxonomy results. Additionally, we applied a taxonomic annotation pipeline to the viral sequences as described in Priest et al. (2023) (13). Viral host predictions were performed using iPHoP v.1.4.2 (14), with all bacterial and archaeal GTDB-Tk-classified MAGs from this study added to the iPHoP database (version Jun_2025_pub_rw).

Plasmid abundance across sediment depths was quantified by mapping quality-filtered paired-end metagenomic reads to the dereplicated plasmid reference set using CoverM. Abundance metrics including mean coverage, covered fraction, relative abundance, and RPKM were calculated using alignment thresholds of ≥95% nucleotide identity and ≥85% read alignment. To assess plasmid transcriptional activity, paired-end metatranscriptomic reads were mapped to the same dereplicated plasmid reference set using CoverM, and transcript abundance was quantified per plasmid using TPM.

**Community-level analysis: Estimation of abundance and transcription of genes, MAGs, and viral contigs**

To enable comparison of microbial community gene content across samples, we constructed a non-redundant gene catalogue. Genes were predicted from all metagenomic contigs using pyrodigal-GV v.0.3.2 (15) and clustered at 95% nucleotide identity with a minimum of 90% alignment coverage using the easy-linclust workflow in MMSeqs2 v18.8cc5c (16). Representative sequences from each cluster were retained to generate the non-redundant gene catalogue. Functional annotation of the catalogue was performed with hmmsearch v.3.4 (17) using the gathering cutoff threshold (--cut_ga) against the KEGG Orthology database (accessed March 2025) and dbCAN (version v14), with the best-scoring annotation per gene cluster selected based on bitscore and e-value.

**Co-occurrence network**

Co-occurrence networks were constructed based on the log₂-transformed ratio of metatranscriptomic to metagenomic abundance [log₂(∑MT/MG)]. Pairwise associations between taxa were quantified using Spearman’s rank correlation. Only strong correlations were retained (|ρ| ≥ 0.7). To limit network density, the three strongest edges per node were selected for downstream analyses and visualization. Taxa were further filtered to include only those detected in at least five samples (prevalence ≥ 5) and exhibiting sufficient variability across samples (standard deviation ≥ 0.3).

**Results and Discussion**

**Description of recovered MAGs**

Other abundant bacterial MAGs included Patescibacteria (n = 126), Planctomycetota (n = 67), Desulfobacterota (n = 52), and Chloroflexota, while WOR-3 and Atribacteriota showed increased representation in deeper layers (**Figure S5**). Planctomycetota and Bipolaricaulota also contributed substantially to the transcriptomic signal. Notably, while Patescibacteria represented the most diverse phylum in terms of MAG recovery, they accounted for a relatively small proportion of total transcripts compared to other dominant taxa, in line with the observations on the metagenomic read level.

DHVEG-1 archaea are commonly inferred to be anaerobic heterotrophs, capable of degrading organic matter or fermentation products and utilizing molecular H_2_ (18,19). Their consistent high expression in the Cadagno sediments (**Table S6**) indicates an active role rather than passive persistence. In contrast, Nanoarchaeota and Microarchaeota (members of DPANN superphyla) MAGs, despite **comprising ~1% of the community**, contributed comparatively little to transcription, mirroring the pattern observed for their bacterial counterparts in the Patescibacteria (part of Candidate Phyla Radiation – CPR) (20) and in the total community analysis (**Figure 1A**). This consistent abundance-activity decoupling across both bacterial and archaeal ultrasmall-genome lineages reflects either an episymbiotic lifestyle or dormancy state, or a viral top-down regulation.

**mOTUs and SingleM discrepancies**

The two methods produced discrepant results in two aspects. First, SingleM recovered a comparatively uniform community structure across all depths, whereas mOTUs detected clear depth-dependent shifts. This discrepancy likely reflects differences in marker-gene selection and database composition. The depth gradient observed by mOTUs is more consistent with the expected decline in labile organic matter inputs with depth, which would promote shifts in heterotrophic community structure. Second, SingleM profiling recovered a striking signal from Patescibacteriota (CPR) and Nanoarchaeota (DPANN), which together comprised 5.6–16.9% and 4.3–8.0% of the community, respectively, across all samples and displayed remarkable taxonomic diversity - spanning 126 orders and 775 genera for Patescibacteriota and 366 genera for Nanoarchaeota. Despite this high genomic abundance and diversity, both groups contributed little to the metatranscriptomic signal. Whether this reflects true dormancy, episymbiotic lifestyles with low transcriptional activity, or methodological artifacts related to small genome size remains an open question and is revisited in the context of viral activity.

**References**

1.	Li H, Handsaker B, Wysoker A, Fennell T, Ruan J, Homer N, et al. The Sequence alignment/map format and SAMtools. Bioinformatics. 2009;25(16):2078–9. doi:10.1093/bioinformatics/btp352

2.	Kang DD, Li F, Kirton E, Thomas A, Egan R, An H, et al. MetaBAT 2: an adaptive binning algorithm for robust and efficient genome reconstruction from metagenome assemblies. PeerJ. 2019;7:e7359. doi:10.7717/peerj.7359

3.	Wu YW, Simmons BA, Singer SW. MaxBin 2.0: An automated binning algorithm to recover genomes from multiple metagenomic datasets. Bioinformatics. 2016;32(4):605-7. doi:10.1093/bioinformatics/btv638

4.	Alneberg J, Bjarnason BS, Bruijn ID, Schirmer M, Quick J, Ijaz UZ, et al. Binning metagenomic contigs by coverage and composition. Nat Methods. 2014: 11(11):1144-6. doi:10.1038/nmeth.3103

5.	Sieber CMK, Probst AJ, Sharrar A, Thomas BC, Hess M, Tringe SG, et al. Recovery of genomes from metagenomes via a dereplication, aggregation and scoring strategy. Nat Microbiol. 2018;3(7):836–43. doi:10.1038/s41564-018-0171-1

6.	Olm MR, Brown CT, Brooks B, Banfield JF. dRep: a tool for fast and accurate genomic comparisons that enables improved genome recovery from metagenomes through de-replication. ISME J. 2017 ;11(12):2864–8. doi:10.1038/ismej.2017.126

7.	Bowers RM, Kyrpides NC, Stepanauskas R, Harmon-Smith M, Doud D, Reddy TBK, et al. Minimum information about a single amplified genome (MISAG) and a metagenome-assembled genome (MIMAG) of bacteria and archaea. Nat Biotechnol. 2017;35(8):725–31. doi:10.1038/nbt.3893

8.	Chaumeil PA, Mussig AJ, Hugenholtz P, Parks DH. GTDB-Tk: a toolkit to classify genomes with the Genome Taxonomy Database. Hancock J, editor. Bioinformatics. 2019;36(6):1925–7. doi:10.1093/bioinformatics/btz848

9.	Aroney STN, Newell RJP, Nissen JN, Camargo AP, Tyson GW, Woodcroft BJ. CoverM: read alignment statistics for metagenomics. Bioinformatics. 2025;41(4):btaf147. doi:10.1093/bioinformatics/btaf147

10.	Shaffer M, Borton MA, McGivern BB, Zayed AA, La Rosa SL, Solden LM, et al. DRAM for distilling microbial metabolism to automate the curation of microbiome function. Nucleic Acids Res. 2020;48(16):8883–900. doi:10.1093/nar/gkaa621

11.	Zhou Z, Tran PQ, Breister AM, Liu Y, Kieft K, Cowley ES, et al. METABOLIC: high-throughput profiling of microbial genomes for functional traits, metabolism, biogeochemistry, and community-scale functional networks. Microbiome. 2022;10(1):33. doi:10.1186/s40168-021-01213-8

12.	Dede B, Reeves EP, Walter M, Bach W, Amann R, Meyerdierks A. Bacterial chemolithoautotrophy in ultramafic plumes along the Mid-Atlantic Ridge. ISME J. 2024 ;18(1):wrae165. doi:10.1093/ismejo/wrae165

13.	Priest T, Vidal-Melgosa S, Hehemann JH, Amann R, Fuchs BM. Carbohydrates and carbohydrate degradation gene abundance and transcription in Atlantic waters of the Arctic. ISME Commun. 2023;3(1):130. doi:10.1038/s43705-023-00324-7

14.	Roux S, Camargo AP, Coutinho FH, Dabdoub SM, Dutilh BE, Nayfach S, et al. iPHoP: An integrated machine learning framework to maximize host prediction for metagenome-derived viruses of archaea and bacteria. PLOS Biol. 2023;21(4):e3002083. doi:10.1371/journal.pbio.3002083

15.	Larralde M. Pyrodigal: Python bindings and interface to Prodigal, an efficient method for gene prediction in prokaryotes. J Open Source Softw. 2022;7(72):4296. doi:10.21105/joss.04296

16.	Mirdita M, Steinegger M, Söding J. MMseqs2 desktop and local web server app for fast, interactive sequence searches. Bioinformatics. 2019;35(16):2856–8. doi:10.1093/bioinformatics/bty1057

17.	Eddy SR. Accelerated profile HMM searches. PLoS Comput Biol. 2011;7(10): e1002195. doi:10.1371/journal.pcbi.1002195

18.	Zhang X, Huang Y, Liu Y, Xu W, Pan J, Zheng X, et al. An Ancient Respiratory System in the Widespread Sedimentary Archaea Thermoprofundales. Mol Biol Evol. 2022;39(10):msac213. doi:10.1093/molbev/msac213

19.	Zheng PF, Wei Z, Zhou Y, Li Q, Qi Z, Diao X, et al. Genomic Evidence for the Recycling of Complex Organic Carbon by Novel Thermoplasmatota Clades in Deep-Sea Sediments. mSystems. 2022;7(3):e00077-22. doi:10.1128/msystems.00077-22

20.	He C, Keren R, Whittaker ML, Farag IF, Doudna JA, Cate JHD, et al. Genome-resolved metagenomics reveals site-specific diversity of episymbiotic CPR bacteria and DPANN archaea in groundwater ecosystems. Nat Microbiol. 2021;6(3):354–65. doi:10.1038/s41564-020-00840-5
